# Supplementary material for: Modulation of the ETV6::RUNX1 Gene Fusion Prevalence in Newborns by Corticosteroid Use During Pregnancy
Source: Int J Mol Sci. 2025 Mar 25;26(7):2971. doi: 10.3390/ijms26072971 (PMC11988504; doi:10.3390/ijms26072971)
Supplement: Supplementary file 1 [file ijms-26-02971-s001.zip › ijms-3510872-supplementary.pdf]

Supplementary material

TABLE S1: Cord bloods identified as *ETV6::RUNX1*+ by GIPFEL screening (n = 48 of 741). Used GIPFEL primers according to Fueller et al.{Fueller, 2014 #274} (forward, fwd and reverse, rev), frequency of *ETV6::RUNX1+* cells within the sample according to *RUNX1* wildtype control and intervention group are given. In two cases two different *ETV6::RUNX1* fusions were detected.

Nr.	fwd	rev	Frequency	fwd	rev	Frequency	Group
1	12	2	9.8 x 10-3				Control
2	13	3	1.9 x 10-3				Control
3	24	3	5.3 x 10-3				Control
4	12	3	1.2 x 10-2				Control
5	11	3	1.5 x 10-3				Control
6	27	1	1.5 x 10-3				Control
7	14	3	3.3 x 10-3				Control
8	20	3	1.3 x 10-2				Control
9	15	3	4.9 x 10-3				Control
10	22	3	1.3 x 10-3				Control
11	10	1	2 x 10-3				Control
12	18	3	2,5 x 10-3				Control
13	4	3	3,9 x 10-3				Control
14	15	3	2,3 x 10-3				Control
15	17	3	6,7 x 10-3				Control
16	11	3	1,1 x 10-3				Control
17	4	2	7,6 x 10-3				Control
18	14	3	3.0 x 10-3				Mindfulness
19	1	2	1.6 x 10-2				Mindfulness
20	29	3	5.0 x 10-2				Mindfulness
21	17	1	4.5 x 10-3				Mindfulness
22	23	2	7.0 x 10-3				Mindfulness
23	13	2	9.3 x 10-4				Mindfulness
24	25	3	1.6 x 10-4				Mindfulness
25	10	2	2.8 x 10-3				Mindfulness
26	17	1	1.8 x 10-2				Mindfulness

27	24	3	3.8 x 10-4				Mindfulness
28	29	1	5.6 x 10-2				Mindfulness
29	11	2	1.1 x 10-3	24	2	1.1 x 10-3	Mindfulness
30	14	2	1 x 10-2				Mindfulness
31	4	2	6,3 x 10-3	13	2	2,9 x 10-4	Mindfulness
32	11	2	1,8 x 10-3				Mindfulness
33	23	3	1.2 x 10-3				Nutrition
34	13	1	3.1 x 10-3				Nutrition
35	15	2	1.5 x 10-2				Nutrition
36	23	3	2.2 x 10-3				Nutrition
37	12	3	1.4 x 10-2				Nutrition
38	14	2	4.0 x 10-3				Nutrition
39	8	2	9.6 x 10-4				Nutrition
40	28	2	1.8 x 10-3				Nutrition
41	25	1	1 x 10-3				Nutrition
42	21	2	3,5 x 10-3				Nutrition
43	12	2	3,9 x 10-3				Nutrition
44	26	3	2,4 x 10-3				Nutrition
45	17	2	2,4 x 10-3				Nutrition
46	21	2	1,4 x 10-3				Nutrition
47	17	2	3,2 x 10-3				Nutrition
48	4	2	7,4 x 10-3				Nutrition

TABLE S2 - Maternal and prenatal characteristics of the study participants

	No. (%)
**Maternal baseline characteristics**	
Maternal age (years)	36.8 (5.07)
BMI before pregnancy (kg/m^2)	24.1 (4.71)
Ethnicity	
- White	578 (78.0)
- Black	14 (1.9)
- Asian	16 (2.2)
- Indian	5 (0.7)
- Latin American	119 (16.1)
- Maghreb	9 (1.2)
Study class	
- Primary/no studies	48 (6.5)
- Secondary/tech	256 (34.6)
- University	437 (59.0)
Socioeconomic status$^{\Psi}$	
- High	411 (55.5)
- Medium	282 (38.1)
- Low	48 (6.5)
Nulliparous	426 (57.5)
Pregestational diabetes	43 (5.8)
Thyroid disorder	94 (12.7)
Autoimmune disease	118 (15.9)
Chronic hypertension	30 (4.1)
Chronic kidney disease	18 (2.4)
Obesity$^{\Phi}$	88 (11.9)
**Pregnancy and prenatal outcomes**	
Intervention group	
- Usual care	245 (33.1)
- Stress reduction	246 (33.2)
- Mediterranean Diet	250 (33.7)
Gestational diabetes	84 (11.4)
Maternal cigarette smoking during pregnancy	56 (7.6)

Alcohol intake	17 (2.3)
Recreational drug consumption	3 (0.4)
Folate supplementation intake	563 (76.0)
Exogenous corticosteroids	39 (5.3)
Preterm birth	41 (5.5)
Preeclampsia	57 (7.7)
Small for gestational age[ϒ]	129 (17.4)
Gestational age at delivery (weeks)	39.4 (1.67)
Neonatal sex	
- Female	355 (48.0)
- Male	385 (52.0)

Results are displayed as n (%) or mean (SD).
BMI denotes body mass index.
[Ψ]Socio economic status defined as low if participants reported having never worked or being unemployed for more than 2 years and having a partner with unqualified work or who was unemployed; high if they reported university studies regardless of whether they were working; and medium if any other situations.
[Φ]Obesity defined as body mass index above 30.
[ϒ]Small for gestational age defined as birthweight below the 10th centile according to local standards

TABLE S3 – Changes in dietary key-foods intake and Mediterranean Diet adherence evaluated at baseline and final visits according to ETV6::RUNX1 positivity.

		Between-group changes			
		**Neonatal ETV6::RUNX1 + (N=36)**	**Neonatal ETV6::RUNX1 - (N=544)**		
				*p*c	*OR T3 vs T1 (95% CI)*
Extra Virgin Olive Oil – g/d	Baseline[a]	32.0 (17.2)	33.4 (19.2)	0.485	NA
	Final[b]	44.8 (2.62)**	40.1 (0.67)**	0.121	NA
Total nuts – g/d	Baseline[a]	19.0 (21.6)	18.5 (18.9)	0.198	0.79 (0.37 to 1.69)
	Final[b]	23.8 (3.14)	24.5 (0.79)**	0.913	1.11 (0.48 to 2.57)
Vegetables – g/d	Baseline[a]	270.6 (103.4)	287.4 (124.0)	0.697	0.69 (0.30 to 1.60)
	Final[b]	303.1 (4.41)	312.5 (16.9)**	0.852	1.26 (0.58 to 2.77)
Legumes – g/d	Baseline[a]	54.5 (35.4)	52.8 (41.0)	0.787	1.05 (0.50 to 2.19)
	Final[b]	58.9 (6.88)	63.4 (1.75)**	0.778	1.24 (0.55 to 2.78)
Fruits – g/d	Baseline[a]	366.4 (218.3)	325.0 (170.8)	0.355	1.17 (0.52 to 2.63)
	Final[b]	333.7 (27.6)	357.8 (7.22)**	0.585	0.49 (0.20 to 1.16)
Refined cereals – g/d	Baseline[a]	75.8 (47.2)	64.2 (42.4)	0.221	1.23 (0.55 to 2.79)
	Final[b]	59.5 (6.29)	48.0 (1.62)**	0.202	2.03 (0.89 to 4.64)
Whole grain cereals – g/d	Baseline[a]	25.7 (24.9)	37.0 (39.7)	**0.050**	0.51 (0.21 to 1.28)
	Final[b]	49.4 (6.30)*	48.6 (1.62)**	0.955	0.57 (0.24 to 1.34)
Fish or seafood – g/d	Baseline[a]	71.9 (33.9)	72.7 (40.4)	0.946	1.07 (0.44 to 2.58)
	Final[b]	84.8 (6.55)*	83.6 (1.72)**	0.909	1.26 (0.53 to 2.98)

Fat fish – g/d	Baseline[a]	14.2 (11.8)	14.7 (16.1)	0.912	0.47 (0.11 to 2.09)
	Final[b]	24.3 (3.14)**	22.8 (0.81)**	0.595	1.38 (0.56 to 3.39)
Red meat – g/d	Baseline[a]	51.7 (36.9)	47.9 (33.6)	0.805	1.24 (0.54 to 2.87)
	Final[b]	46.7 (4.48)	44.8 (1.15)*	0.849	0.91 (0.39 to 2.11)
Processed meat – g/d	Baseline[a]	26.3 (22.3)	34.2 (27.7)	0.221	0.50 (0.21 to 1.16)
	Final[b]	26.0 (3.15)	31.5 (0.81)*	0.227	0.48 (0.20 to 1.18)
Pastries, cakes, or sweets – g/d	Baseline[a]	44.0 (26.7)	39.0(36.3)	0.543	2.48 (1.10 to 5.61)
	Final[b]	37.5 (4.78)	35.2 (1.22)*	0.440	1.18 (0.52 to 2.67)
Dairy products – g/d	Baseline[a]	345.0 (228.7)	332.9 (203.6)	0.947	1.19 (0.54 to 2.64)
	Final[b]	437.9 (34.0)**	398.6 (8.72)**	0.458	1.87 (0.83 to 4.19)
Mediterranean diet adherence - points	Baseline[a]	7.39 (2.15)	7.67 (2.49)	0.161	0.44 (0.14 to 1.35)
	Final[b]	10.1 (0.46)**	9.35 (0.11)**	0.061	1.23 (0.50 to 3.02)

[a]Baseline values are observed means (SD). [b]Final values are baseline-adjusted (least-squares) means (SE) and comparison among groups done with ANCOVA analysis. *P<0.05 and **P<0.001 final from baseline comparison. [c]ANCOVA analysis

TABLE S4 – Changes in nutrients intake evaluated at baseline and final visits according to ETV6::RUNX1 positivity

		Between-group changes			
		**Neonatal ETV6::RUNX1 + (N=36)**	**Neonatal ETV6::RUNX1 - (N=544)**		
				P^{c}	*OR T3 vs T1 (95% CI)*
Energy – kcal/d	Baseline[a]	2446 (640.5)	2437 (504.3)	0.357	0.92 (0.44 to 1.93)
	Final[b]	2564 (70.0)	2508 (18.0)*	0.625	2.43 (0.92 to 6.47)
Protein – kcal/d	Baseline[a]	99.7 (27.2)	103.7 (24.4)	0.591	0.55 (0.23 to 1.34)
	Final[b]	110.8 (3.83)	109.9 (0.98)**	0.619	1.09 (0.52 to 2.31)
Carbohydrate – g/d	Baseline[a]	231.5 (78.1)	218.2 (58.9)	0.305	1.22 (0.51 to 2.93)
	Final[b]	222.1 (7.68)	215.2 (1.97)	0.669	2.31 (0.86 to 6.20)
Fiber – g/d	Baseline[a]	33.8 (11.0)	32.9 (10.7)	0.136	1.13 (0.53 to 2.44)
	Final[b]	34.9 (1.51)	35.2 (0.39)**	0.619	1.65 (0.70 to 3.90)
Total fat – g/d	Baseline[a]	124.5 (35.0)	127.5 (30.4)	0.150	0.97 (0.44 to 2.15)
	Final[b]	137.1 (4.30)*	133.9 (1.10)**	0.542	1.96 (0.76 to 5.02)
SFA – g/d	Baseline[a]	34.1 (10.3)	34.5 (10.1)	0.374	0.98 (0.42 to 2.27)
	Final[b]	35.1 (1.40)	35.8 (0.36)*	0.655	0.96 (0.39 to 2.35)
MUFA – g/d	Baseline[a]	59.4 (16.6)	61.4 (15.5)	0.184	1.08 (0.47 to 2.52)
	Final[b]	67.0 (2.12)*	64.3 (0.54)**	0.308	1.72 (0.70 to 4.24)
PUFA – g/d	Baseline[a]	21.7 (9.42)	22.1 (7.71)	0.215	0.95 (0.46 to 1.96)
	Final[b]	24.7 (1.22)	24.0 (0.31)**	0.847	1.44 (0.63 to 3.33)
-Linoleic acid – g/d	Baseline[a]	14.6 (6.71)	14.5 (5.83)	0.495	0.93 (0.44 to 1.96)

	Final[b]	16.4 (0.90)	15.7 (0.23)**	0.651	1.43 (0.64 to 3.18)
- Linolenic acid – g/d	Baseline[a]	1.46 (0.74)	1.38 (0.59)	0.203	0.94 (0.43 to 2.02)
	Final[b]	1.72 (0.12)	1.70 (0.03)**	0.961	1.35 (0.62 to 2.97)
EPA – g/d	Baseline[a]	0.15 (0.08)	0.15 (0.10)	0.972	1.05 (0.47 to 2.35)
	Final[b]	0.21 (0.02)**	0.20 (0.00)**	0.925	1.25 (0.51 to 3.09)
DHA – g/d	Baseline[a]	0.32 (0.17)	0.32 (0.25)	0.992	1.06 (0.44 to 2.56)
	Final[b]	0.46 (0.05)**	0.44 (0.01)**	0.695	1.63 (0.66 to 4.03)
Trans-FA – g/d	Baseline[a]	1.60 (1.04)	1.69 (1.17)	0.864	0.96 (0.45 to 2.07)
	Final[b]	1.75 (0.15)	1.46 (0.04)**	0.126	1.47 (0.67 to 3.21)
Cholesterol – mg/d	Baseline[a]	312.0 (98.4)	331.4 (97.4)	0.508	0.71 (0.32 to 1.58)
	Final[b]	336.2 (13.5)	340.7 (3.47)*	0.217	0.67 (0.29 to 1.56)

SFA denotes Saturated fatty acids. MUFA Monounsaturated fatty acids. PUFA Polyunsaturated fatty acids. EPA Eicosapentaenoic acid. DHA Docosahexaenoic acid and FA Fatty acids. [a]Baseline values are observed means (SD). [b]Final values are baseline-adjusted (least-squares) means (SE) and comparison among groups done with ANCOVA analysis. *P<0.05 and **P<0.001 final from baseline comparison. [c]ANCOVA analysis.

TABLE S5– Dose-response effect of bethamethasone on neonatal ETV6::RUNX1 positivity

**Number of betamethasone doses**	Neonatal ETV6::RUNX 1 +	Total of patients receiving bethamethasone	Estimated probability	
1	0	3	0	
2	5	21	0.238095	
3	0	1	0	
				P for linear trend= 0.6171
